# *Bifidobacterium longum* supplementation improves age‐related delays in fracture repair

**DOI:** 10.1111/acel.13786

**Published:** 2023-01-27

**Authors:** Joseph L. Roberts, Mateo Golloshi, Derek B. Harding, Madison Conduah, Guanglu Liu, Hicham Drissi

**Affiliations:** ^1^ Department of Orthopaedics Emory University School of Medicine Atlanta Georgia USA; ^2^ Atlanta VA Health Care System Decatur Georgia USA

**Keywords:** aging, *Bifidobacterium longum*, femur fracture, gut microbiome, probiotics

## Abstract

Age‐related delays in bone repair remains an important clinical issue that can prolong pain and suffering. It is now well established that inflammation increases with aging and that this exacerbated inflammatory response can influence skeletal regeneration. Recently, simple dietary supplementation with beneficial probiotic bacteria has been shown to influence fracture repair in young mice. However, the contribution of the gut microbiota to age‐related impairments in fracture healing remains unknown. Here, we sought to determine whether supplementation with a single beneficial probiotic species, *Bifidobacterium longum* (*B. longum*), would promote fracture repair in aged (18‐month‐old) female mice. We found that *B. longum* supplementation accelerated bony callus formation which improved mechanical properties of the fractured limb. We attribute these pro‐regenerative effects of *B. longum* to preservation of intestinal barrier, dampened systemic inflammation, and maintenance of the microbiota community structure. Moreover, *B. longum* attenuated many of the fracture‐induced systemic pathologies. Our study provides evidence that targeting the gut microbiota using simple dietary approaches can improve fracture healing outcomes and minimize systemic pathologies in the context of aging.

## Graphical abstract

Dietary supplementation with the probiotic species *Bifidobacterium longum* (*B. longum*) accelerated and enhanced femoral fracture repair in aged mice as well as attenuated many of the systemic consequences of fracture. *Bifidobacterium longum* supplementation prevented gut leakiness, dampened inflammation, and maintained the community structure of the gut microbiota throughout bone repair.

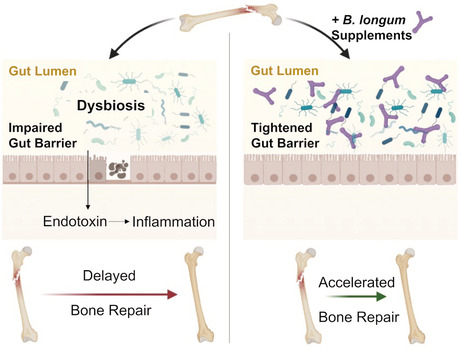

## 1 INTRODUCTION

Normal aging is associated with a range of physiologic changes that affect nearly every tissue, including bone. The rate of bone resorption is accelerated in aging that results in weaker bones that are more prone to fractures (Demontiero et al., 2012). Unfortunately, aged individuals experience a natural decline in healing potential, which leads to significant delays in bone healing that prolongs patient suffering (Nieminen et al., 1981; Nikolaou et al., 2009). Fracture healing is also delayed in aged mice (Clark et al., 2020; Clement et al., 2022; Liu et al., 2022; Naik et al., 2009) and these fractures induce exacerbated systemic responses compared to young mice, including increased inflammation (Emami et al., 2019), post‐traumatic bone loss (Ely et al., 2020; Emami et al., 2019), and pain (Majuta et al., 2018). Identifying novel strategies that can accelerate bone repair while simultaneously attenuating many of the fracture‐induced sequalae is of special interest to the elderly population.

Aging is also associated with considerable taxonomic changes in the gut microbiota community structure that is associated with frailty (Langille et al., 2014; Shin et al., 2021). Traumatic injuries, including fracture, can also alter the composition of the intestinal microbiota, which has emerged as an important contributor to musculoskeletal health and disease (Li et al., 2021; Steves et al., 2016; Vitetta et al., 2013). A common strategy to transiently influence the microbiota community structure is through dietary supplementation with live bacteria called probiotics, that when consumed in adequate amounts confer a health benefit (Tyagi et al., 2018; Wieërs et al., 2019). Most probiotic species belong to the *Lactobacillus* and *Bifidobacterium* genera, several of which have reported bone‐protective properties in the context of rapid bone turnover (Collins et al., 2017). These beneficial effects are often attributed to modulation of inflammation and intestinal function (Collins et al., 2017). More recently, the beneficial role of probiotics during bone repair has been investigated. We reported that supplementing young mice with the probiotic species *Bifidobacterium adolescentis* accelerated cartilaginous callus remodeling and attenuated many of the systemic responses to femoral fracture, including intestinal leakiness and inflammation (Roberts et al., 2020). Similarly, a subsequent report demonstrated that supplementing young mice with the probiotic species *Akkermansia muciniphila* or *Lactobacillus gasseri* can promote bone healing through a similar mechanism of reduced gut permeability and inflammation (Liu et al., 2020). A beneficial effect of probiotics during recovery from fractures has also been reported in elderly patients consuming *Lactobacillus casei* Shirota that experienced significantly improved pain and functional outcomes throughout recovery from distal radius, humerus, and rib fractures (Lei et al., 2016, 2018; Zhang et al., 2019).

*Bifidobacterium longum* (*B. longum*) is a Gram‐positive, anaerobic pioneer species and the most abundant *Bifidobacterium* species within the gastrointestinal tract of healthy infants and adults (Ouwehand et al., 2008; Turroni et al., 2012). Many health‐promoting effects have been ascribed to *B. longum*, including improving gut function and decreasing intestinal permeability (Chen et al., 2011; Pitkala et al., 2007), decreasing inflammation (Chen et al., 2011; MacSharry et al., 2012; Ouwehand et al., 2008; Sapra et al., 2022), preventing ovariectomy‐induced bone loss (Montazeri‐Najafabady et al., 2021; Parvaneh et al., 2015; Sapra et al., 2022), and influencing the diversity of the microbiome (Ouwehand et al., 2008). Herein, we investigated whether supplementation with the commonly consumed probiotic species *B. longum* would accelerate fracture healing in aged mice that display a natural delay in secondary bone repair and attenuate post‐traumatic sequelae. We demonstrate that supplementation with a single probiotic species *B. longum* accelerated fracture healing, indicated by increased callus bone and improved biomechanical properties, and protected the intact skeleton from post‐traumatic bone loss. These beneficial effects likely stemmed from preservation of intestinal function, dampened inflammation, and inhibition of fracture‐induced dysbiosis.

## 2 METHODS

### 2.1 Animals

Seventeen‐month‐old female C57BL/6JN mice were obtained from the National Institutes of Aging and allowed to acclimate to housing conditions for 1 month. Mice had ab libitum access to sterilized (irradiated) extruded chow diet (Envigo #2918) and water (0.1 micron filtered). This diet contains 18% protein (24% of calories), 44% available carbohydrate (58% of calories), and 6% fat (18% calories) equating to 3.1 kcal/g. All mice were group housed (*n* = 3–5/cage) in the Atlanta Veterans Affairs Medical Center (VAMC) vivarium in specific pathogen‐free cages that contained corncob bedding and nestlets and in controlled conditions (temperature: 21–24°C; humidity: 40%–70%; light/dark cycle: 12/12 h). During this acclimation period, the bedding was repeatedly exchanged between cages to minimize cage effects on the microbiota community structure. One week prior to gavage initiation, mice were randomly switched between cages. All experiments were performed in accordance with the NIH Guide for the Care and Use of Laboratory Animals and were approved by the Atlanta VAMC Institutional Animal Care and Use Committee (Approval #V017‐19).

### 2.2 Bacterial culture

*Bifidobacterium longum* subsp. *longum Reuter* (*B. longum*, ATCC #15707) was cultured under anaerobic conditions in reinforced clostridial media containing 9% Oxyrase (Sigma‐Aldrich #SAE0013) at 37°C for approximately 24 h. Each day prior to gavage, the bacteria were centrifuged at 3000 × g, media aspirated, and washed in sterile PBS for a total of two times. The pellet was resuspended in sterile PBS and used for gavage.

### 2.3 Probiotic treatment

After the 1‐month acclimation period, the mice were randomly assigned to receive either sterile PBS (100 μl; Life Technologies) or 1 × 10^8^ – 1 × 10^9^ CFU of *B. longum* through oral gavage once per day. After 2 weeks of supplementation, one cohort of mice was randomly selected to be killed prior to fracture and referred to as day 0 (or baseline), while the remaining mice were subjected to a fracture as described below. All mice continued to receive their assigned treatment for 3, 7, 14, 21. or 35 days postfracture. Mice were weighed daily using a calibrated balance (*n* = 10/group).

### 2.4 Fracture model

Femoral fractures were generated as we had previously described (Paglia et al., 2020; Roberts et al., 2021). Briefly, mice were anesthetized with isoflurane, administered analgesic (Buprenorphine SR), and the left hind limb shaved and cleaned with chlorohexidine and isopropanol. The articular surface of the femoral intercondylar notch was then perforated with a 25‐gauge needle through the skin, followed by insertion of a sterilized stainless steel 316LVM wire (diameter 0.16 inch) into the medullary canal. A transverse mid‐diaphyseal fracture was then created using 3‐point bending via a blunt guillotine device. The fractured limbs were radiographically examined by digital X‐ray (Bruker) immediately postfracture to confirm fracture location and pin placement. Animals with severely comminuted, distal, or proximal fractures were excluded from the histologic and μCT studies, but were used for other outcomes to assess systemic effects of fracture. Mice were allowed to fully weight‐bear after recovery from anesthesia.

### 2.5 Microcomputed tomography

Micro‐computed tomography (μCT) was performed on the fractured femur and L3 vertebrae using a μCT40 scanner (Scanco Medical AG) using an isotropic voxel size of 6 μm (70 kVp and 114 mA, integration time of 200 ms). Bones were fixed for 1 week in 10% neutral buffered formalin at 4°C followed by scanning in PBS medium. For fracture callus analyses, the entire callus was manually segmented to exclude existing cortical bone and any bone fragments. The following measures of callus structure and composition were quantified for each fracture callus at day 14 (*n* = 5–7/group) and day 21 (*n* = 6–9/group): total callus volume (TV); mineralized callus volume (BV); and bone volume fraction (BV/TV). The trabecular bone within the L3 vertebral body from the cranial to caudal growth plate was determined ex vivo using the auto contour function. The following measures of vertebral body trabecular bone structure and composition were quantified from approximately 350 slices (*n* = 7–8/group): bone volume fraction (BV/TV); trabecular thickness (Tb.Th); trabecular number (Tb.N); bone surface density (BS/BV); and structure model index (SMI).

### 2.6 Histology and static histomorphometry

Fractured femora (*n* = 4–5/group) were fixed in 10% neutral buffered formalin for 1 week at 4°C, then decalcified in 14% EDTA (pH 7.2) for 3–4 weeks at room temperature with gentle shaking. The decalcified femora were then dehydrated, embedded in paraffin, and sectioned (7 μm thickness). Histologic sections were then rehydrated through a graded series of alcohols and stained with Safranin O/Fast Green to visualize cartilage and bone. Callus size, callus cartilage, and callus bone were quantified using Osteomeasure (Osteometrics). The percentage of cartilage and bone with the fracture callus was determined by normalizing the tissue to the total size of the callus.

### 2.7 Dual energy X‐ray absorptiometry (DXA) analyses

Mice (*n* = 10/group) were anesthetized using isoflurane inhalation. Bone mineral density and bone mineral content of lumbar spine (L1–L5) were longitudinally determined using a Kubtec 4‐parameter cabinet X‐ray system (40 KV; 1000 μA) using the Kubtec DIGIMUS BMD analysis software prior to fracture, and at day 7, 14, 21, and 35 days postfracture.

### 2.8 Torsion biomechanics

At day 35 postfracture, fractured femora (*n* = 8–9/group) were cleaned of all soft tissue, wrapped in cold PBS‐soaked gauze, and stored at −20°C until time of testing. On the day of testing, the femora were thawed to room temperature, the intramedullary pin removed, and the distal and proximal ends potted in 1 cm^2^ aluminum cubes filled with polymethyl methacrylate (Stoelting Co.). The gauge length, anteroposterior, and mediolateral diameter of each bone was measured using calipers. Torsion biomechanical testing was conducted using a calibrated Instron 68‐SC equipped with a 5 Nm load cell. Torsion was applied at 0.5 N‐M/min to the proximal end of the bone until failure. Data were analyzed using a custom code in MATLAB (MathWorks r2021a). Biomechanical properties of maximum torque to failure, stiffness, rigidity, strength, and energy at failure were calculated from the data as previously described (Díaz‐Hernández et al., 2022).

### 2.9 Radiographic scoring

Qualitative analysis was performed on radiographs of fractures at day 35 postfracture (*n* = 8–9/group) by two independent reviewers. X‐rays were acquired at time of killing using Bruker in‐vivo Xtreme 4MP imaging system. Radiographs were coded and observers were blinded to treatment group. Using a validated 3‐category radiographic scoring system, fracture healing was assessed by: (i) periosteal and endosteal reaction, (ii) callus opacity, and (iii) cortical remodeling and bridging (Roberts et al., 2021). Periosteal and endosteal reaction was determined using a 4‐point scoring system (score: 0–3) to evaluate bridging across the fracture site. Callus opacity was determined using a 4‐point scoring system (score: 0–3) to evaluate mineralization of the callus. Cortical remodeling and bridging was determined using a 5‐point scoring system (score: 0–4) to determine the number of visible cortices and if the medullary canal was well demarcated.

### 2.10 Gene expression

An approximately 1 cm section of the small intestines corresponding to the duodenum and proximal colon was collected, flash frozen, and stored at −80°C until analysis. The tissues were homogenized and total RNA was isolated using TRIzol (Invitrogen) (*n* = 4–5/group/timepoint). First‐strand cDNA was synthesized with oligo(dT) and random primers using qScript cDNA SuperMix (Quantabio). All qRT‐PCR were performed on an Analytik Jena qTower^3^ G Real‐Time PCR Detection System using PerfeCTa SYBR Green FastMix (Quantabio). Amplicon authenticity was confirmed by melt curve analysis. Primer sequences are provided in Table S1 and β‐actin was used as the normalization control. The data were analyzed using the ΔΔCT method by comparing expression levels of genes to baseline (prior to fracture) values within each treatment group.

### 2.11 Sera analyses

Mice were anesthetized using isoflurane and whole blood was obtained by cardiac puncture at time of harvest, allowed to clot at room temperature for ≥30 min, then centrifuged at 10,000 *g* for 10 min. Systemic cytokines were assayed using a Meso Scale Discovery U‐Plex electrochemiluminescence assay for 16 cytokines and chemokines according to manufacturer's instructions at the Emory Multiplex Immunoassay Core using sera obtained at day 3 (*n* = 5/group/timepoint) and day 7 postfracture (*n* = 6/group/timepoint). The investigated markers were EPO, GM‐CSF, IFN‐γ, IL‐1β, IL‐6, IL‐10, IL‐16, IL‐17a, IL‐21, IL‐22, IL‐23, IL‐33, MCP‐1, SDF‐1α, TNF‐α, and VEGF‐a. Undetectable concentrations of any factor were recorded as one half of the lower limit of quantification, with the exception of IL‐23 and IL‐21 which were not detected in more than half of the samples and were excluded from analyses. Lipopolysaccharide binding protein (LBP; Abcam #ab269542) and lipocalin‐2 (LCN2; Abcam #ab199083) were assayed using enzyme‐linked immunosorbent assay kits according to the manufacturer's recommendations (*n* = 4–5/group/timepoint). To assess serum endotoxin levels, serum samples (*n* = 4–6/group/timepoint) were diluted 100‐fold in pyrogen‐free water and heated at 70°C for 15 min. Endotoxin levels were quantified using the Pierce Chromogenic Endotoxin Quant Kit (#A39552; Thermo Scientific) according to the manufacturer's directions.

### 2.12 Gut permeability assay

Mice (*n* = 6–7/group) were fasted for 4 h with access to water and then orally gavaged with 150 μl/mouse of 80 mg/mL fluorescein isothiocyanate–dextran 4 (FITC‐dextran MW: 3–5 kDA; Sigma‐Aldrich #FD4) in sterile PBS (Invitrogen). After an additional 4 h fast without food or water, the tail was cleaned with isopropylalcohol swabs and then nicked using a sterile scalpel blade. Blood was collected using heparinized capillary tubes and plasma was isolated by centrifugation for 10 min at 3000 *g* at 4°C. Plasma FITC‐dextran concentration was determined by measuring the fluorescence at 528 nm with excitation at 485 nm using a Spectramax M2 plate reader (Molecular Devices). Assessment of plasma FITC‐dextran concentrations were assessed longitudinally in the same mice.

### 2.13 Sequencing

Fecal pellets (~50 mg) were longitudinally collected from mice (*n* = 4/group) and stored at −80°C until analysis. DNA extraction, sequencing, and bioinformatics were completed by Microbiome Insights. Briefly, fecal DNA was extracted using the Qiagen MagAttract PowerSoil DNA KF kit. Libraries were prepared using an Illumina Nextera library preparation kit with an in‐house protocol (Illumina). Paired‐end sequencing (150 bp × 2) was completed on a NextSeq 500 in medium‐output mode and sequence reads were processed with the Sunbeam pipeline. Initial quality evaluation was completed using FastQC v0.11.5. Processing took part in four steps: adapter removal, read trimming, low‐complexity‐reads removal, and host‐sequence removals. Adapter removal was done using cutadapt v2.6. Trimming was done with Trimmomatic v0.36 using custom parameters (LEADING:3 TRAILING:3 SLIDINGWINDOW:4:15 MINLEN:36). Low‐complexity sequences were detected with Komplexity v0.3.6. High‐quality reads were mapped to the human genome (Genome Reference Consortium Human Reference 37) and the mouse *Mus musculus* (house mouse) genome assembly GRCm38.p6 and those that mapped to it were with at least 50% similarity across 60% of the read length were removed. The remaining reads were taxonomically classified using Kraken2 with the PlusPF database from 2021‐05‐17.

### 2.14 Statistical and bioinformatic analysis

Results are shown as mean ± SD. Statistical significance was determined by either unpaired two‐tailed Student's *t* test, Mann–Whitney U test, or two‐way ANOVA with Sidak's post hoc testing as indicated in the figure legends using GraphPad Prism software (version 9.4.1). All statistical tests were performed at the 5% significance level. Outliers were identified using the ROUT method (Q = 1%) and were removed from datasets when appropriate. To generate heat maps of serum cytokines, the values were first log‐transformed and then converted to Z‐scores. Microbial alpha diversity was estimated using the Shannon diversity index on raw OTU abundances. Beta diversity was estimated using OTUs to compute the Bray–Curtis indices. Beta diversity was visualized using principal coordinates analysis of ordination (PCoA) and variation in community structure was assessed with permutational multivariate analyses of variance (PERMANOVA). Negative binomial models (DESEq2 R package) for differential abundance testing of taxonomic features were examined for differences between groups across timepoints (Timepoint:Group interaction). *p*‐values were calculated with likelihood ratio tests, with an adjusted *p*‐value = 0.01. Taxa that were present in less than 10% of the samples were removed to reduce false positives. Two‐way ANOVA with Sidak's multiple comparisons test was used to compare changes at all timepoints. All analyses were conducted in the R environment by Microbiome Insights.

## 3 RESULTS

### 3.1 *Bifidobacterium longum* attenuates post‐traumatic body mass loss and lumbar spine bone loss

**FIGURE 1:**
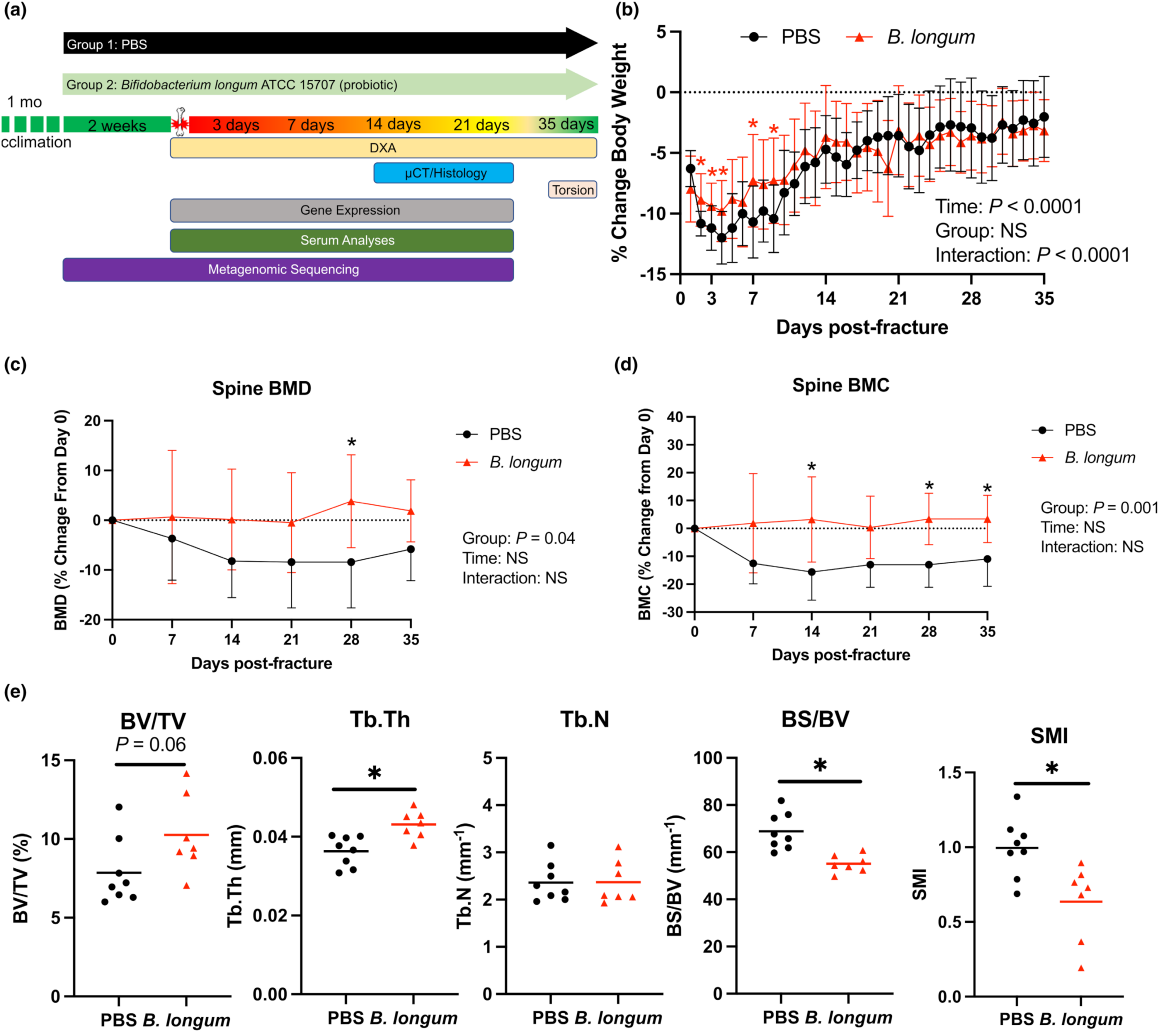
*Bifidobacterium longum* supplementation attenuates post‐traumatic body mass loss and bone loss within the lumbar spine. (a) Study design depicting the random assignment of 18‐month‐old female mice to receive PBS (vehicle control) or *B. longum* (probiotic) for 2 weeks followed by creation of femoral fracture and assessments of healing and systemic effects. (b) *Bifidobacterium longum* supplementation blunted the decrease in body weight during the early post‐fracture period. Data are expressed as percentage change from pre‐fracture (day 0) weight. Two‐way ANOVA followed by Sidak's multiple comparisons testing, *p < 0.05 versus PBS (*n* = 10/group). Changes in *B. longum* lumbar spine (L1–L5) (c) bone mineral density (BMD), and (d) bone mineral content (BMC). Data are expressed as percentage change from pre‐fracture (day 0) values. Two‐way ANOVA followed by Sidak's multiple comparisons testing, *p < 0.05 versus PBS (*n* = 10/group). (e) *Bifidobacterium longum* supplementation increased the trabecular thickness (Tb.Th), bone surface density (BS/BV), and decreased structure model index (SMI) of the trabecular bone within the L3 vertebral body at day 35 post‐fracture. Student's *t* test, *p < 0.05 (*n* = 7‐8/group). Each data point represent an independent observation.

Aged female mice (18‐month‐old) were randomly assigned to receive the candidate probiotic species *B. longum* or vehicle control through a daily oral gavage after a 1‐month acclimation period. After 2 weeks of supplementation, the mice were subjected to a unilateral closed mid‐diaphyseal femoral fracture followed by the assessment of healing and other outcomes at the indicated timepoints postfracture (Figure 1a). The mice continued to receive their respective treatment daily until killing. Prior to fracture (2 weeks of pre‐supplementation), there were no significant differences in body weight, liver weight, or spleen weight between groups (Figure S1a–c). Upon fracture, there was an early and rapid decrease in body weight until day 4 postfracture in both PBS (−12%) and *B. longum* (−9.7%)‐treated mice (Figure 1b); however, *B. longum* significantly attenuated this loss in body weight during the early postfracture period (days 2, 3, 4, 6, and 8). Gross necropsy examination of mice at time of killing did not reveal any histopathologic differences between groups. Additionally, there was a significant increase in liver weight and decrease in spleen weight in *B. longum*‐supplemented mice at day 3 postfracture (Figure S1b,c).

Furthermore, longitudinal DXA analyses of the lumbar spine demonstrated that *B. longum* supplementation protected the lumbar spine from post‐traumatic decreases in bone mineral density (BMD) (Figure 1c) and bone mineral content (BMC) (Figure 1d) observed in the PBS control mice. Micro‐CT analyses of the trabecular bone within the L3 vertebral body demonstrated significantly higher trabecular thickness (Tb.Th) and significantly lower bone surface density (BS/BV) at day 35 postfracture in *B. longum*‐supplemented mice (Figure 1e). Structure model index (SMI) was significantly lower in mice provided the probiotic *B. longum*, which indicates a more plate‐like geometry of trabecular structures (Figure 1e). Together, these data indicate that *B. longum* likely does not have any pathologic effects on aged mice and can attenuate the systemic pathologies induced by femoral fracture.

### 3.2 *Bifidobacterium longum* supplementation accelerates and enhances secondary bone repair

**FIGURE 2:**
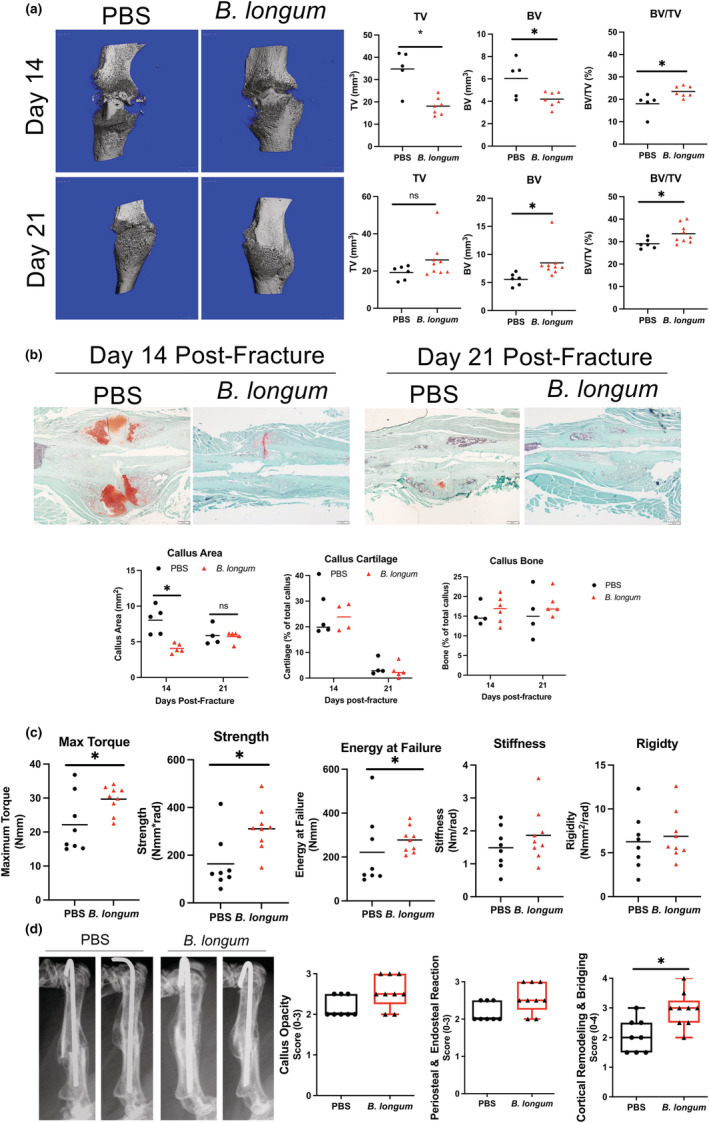
Supplementation with *Bifidobacterium longum* accelerates and enhances bone repair. (a) MicroCT analyses of fracture calluses at day 14 postfracture revealed a significant decrease in callus size (TV) and bone volume (BV), and an increase in callus bone volume fraction (BV/TV) in *B. longum*‐supplemented mice (*n* = 5–7/group). At day 21 postfracture, there was a significant increase in callus BV and BV/TV in mice supplemented with *B. longum* (*n* = 6–9/group). Student's *t* test, *p < 0.05. (b) Histomorphometric analyses showed a decreased callus size at day 14 postfracture in *B. longum*‐supplemented mice. Student's *t* test, *p < 0.05 (*n* = 4–5/group). Outliers were identified using the ROUT method in callus cartilage (excluded value 0.135%). (c) *Bifidobacterium longum* supplementation increased the maximum torque, strength, and energy at failure of fractured bones at day 35 postfracture. Student's *t* test, *p < 0.05 (*n* = 8–9/group). (d) Radiographs of two representative femora from each group at day 35 postfracture and radiographic scoring shows increased cortical bridging and remodeling in *B. longum*‐supplemented mice. Mann–Whitney U test, *p < 0.05 versus PBS (*n* = 7–9/group). Each data point represent an independent observation.

Assessment of callus bone content my micro‐CT revealed that *B. longum* supplemented mice had significantly decreased callus total volume (TV) (−48%) and bone volume (BV) (−31%), which translated into a significant 30% increase in callus bone volume fraction (BV/TV) (Figure 2a) at day 14 postfracture. At day 21 postfracture, no differences in callus TV were observed between groups, but there was a significant increase in callus BV (+53%) and BV/TV (+15%) in mice supplemented with *B. longum* (Figure 2a). Further assessment of callus composition using histomorphometric analyses confirmed the smaller callus size (−49%) in the *B. longum*‐supplemented mice at day 14 postfracture (Figure 2b). However, no significant differences in callus cartilage or bone content were observed between groups at day 14 or 21 postfracture by histomorphometry (Figure 2b).

We next sought to determine if the increased callus bone observed in *B. longum*‐supplemented mice translated into improved mechanical properties using torsion testing, which is the preferred method of assessing strength of fractured bones (Knox et al., 2021). Torsion biomechanical testing of fractured femora at day 35 postfracture revealed an increase in maximum torque (+38%), strength (+81%), and energy at failure (+25%) in mice that received the daily *B. longum* supplement (Figure 2c). No significant differences in stiffness or rigidity were observed between groups. Radiographic scoring of the fracture callus at day 35 postfracture revealed a significant increase in cortical remodeling and bridging in the *B. longum*‐supplemented mice (Figure 2d). Together, these data indicate that *B. longum* supplementation accelerates the time course of fracture healing in aged mice.

### 3.3 *Bifidobacterium longum* maintains the integrity of the intestinal barrier during fracture healing

**FIGURE 3:**
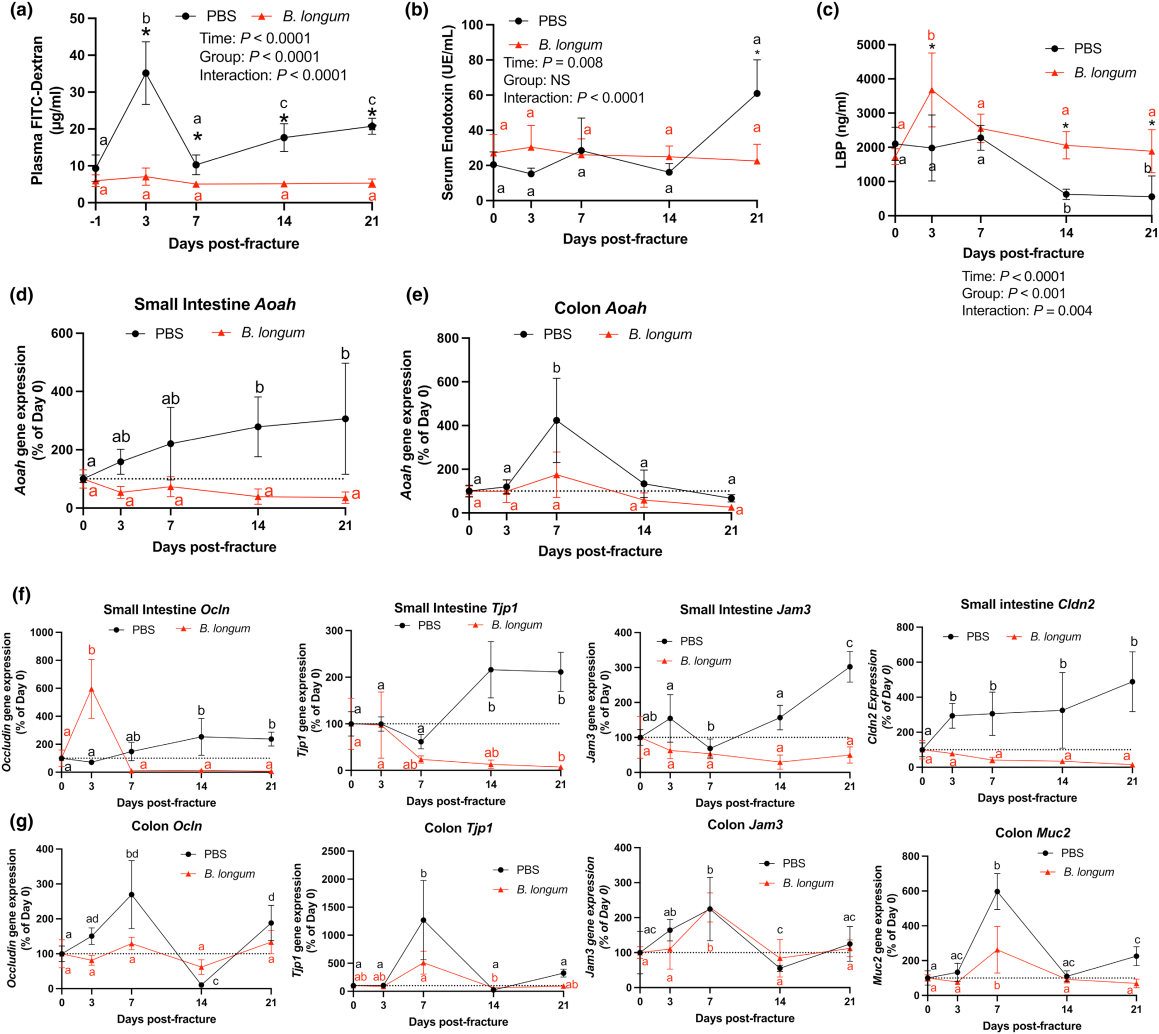
*Bifidobacterium longum* supplementation maintains the integrity of the intestinal barrier. (a) *Bifidobacterium longum* supplementation prevented increase in plasma FITC‐dextran concentrations after fracture. *p < 0.05 versus PBS (*n* = 6–7/group/timepoint). Values of the same color not sharing a common letter are significantly different, *P* < 0.05. (b) Serum endotoxin levels were significantly lower in *B. longum*‐supplemented mice at day 21 postfracture. *p < 0.05 versus PBS (*n* = 4–6/group/timepoint). Values of the same color not sharing a common letter are significantly different, *p* < 0.05. (c) Serum lipopolysaccharide binding protein (LBP) levels were significantly higher in *B. longum*‐supplemented mice at days 3, 14, and 21 postfracture. *p < 0.05 versus PBS. Values of the same color not sharing a common letter are significantly different, *p* < 0.05 (*n* = 4–5/group/timepoint). (d) Small intestine *Aoah* gene expression did not change in the *B. longum*‐supplemented mice but increased at days 14 and 21 postfracture in PBS‐treated mice. (e) Colon *Aoah* gene expression did not change in the *B. longum*‐supplemented mice but increased at day 7 postfracture in the PBS‐treated mice. Changes in (f) small intestine and (g) colon tight junction gene expression compared to baseline no fracture (day 0) shows a differential response to fracture in *B. longum* and PBS‐supplemented mice. Data are expressed as percent change from pre‐fracture (day 0) values. Two‐way ANOVA followed by Sidak's multiple comparison testing, values of the same color not sharing a common letter are significantly different, *p* < 0.05 (*n* = 4–5/group/timepoint).

Probiotics partly influence host physiology by tightening the intestinal barrier, which we and others have reported to be disrupted following traumatic bone injuries in young mice (Liu et al., 2020; Roberts et al., 2020). However, the influence of probiotics on fracture‐induced intestinal permeability within the context of aging remains unknown. Prior to fracture, there were no differences in intestinal permeability between groups. However, closed femoral fracture induced a significant increase in intestinal permeability (+277%; *p* < 0.05) in the PBS‐treated mice at day 3 postfracture as assessed by plasma FITC‐dextran levels (Figure 3a). Conversely, *B. longum* supplementation abrogated this initial fracture‐induced increase in intestinal permeability (+18%; *p* = 0.3), with levels remaining significantly lower than the PBS‐supplemented mice until day 21 postfracture (Figure 3a). No significant differences in serum endotoxin levels were detected during the early postfracture period (days 0–14) between groups or in response to fracture; however, at day 21 postfracture, there was a significant increase in serum endotoxin in the PBS control mice (Figure 3b). *B. longum* supplementation induced a significant increase (+113%) in serum lipopolysaccharide binding protein (LBP) at day 3 postfracture compared to PBS‐treated mice (Figure 3c). Sera LBP returned to baseline (pre‐fracture) by day 7 postfracture in the *B. longum*‐treated mice (Figure 3c). Conversely, in the PBS control mice, serum LBP did not change in the immediate postfracture period but significantly decreased at day 14 and 21 postfracture (Figure 3c).

We next measured gene expression of acyloxyacyl hydrolase (*Aoah*) within the small intestine and the colon, which is responsible for detoxifying endotoxin/LPS (Munford & Hunter, 1992). Within the small intestine, femoral fracture induced a significant increase in *Aoah* gene expression at day 14 and 21 postfracture compared to baseline pre‐fracture levels (Figure 3d). No significant changes in *Aoah* gene expression were observed in small intestine of the *B. longum*‐supplemented mice during the postfracture period (Figure 3d). In the colon, femoral fracture significantly induced *Aoah* gene expression at day 7 postfracture in the PBS control mice, which was not observed in the *B. longum‐*supplemented mice (Figure 3e).

To further decipher the mechanisms through which *B. longum* supplementation influenced intestinal permeability and serum endotoxin levels postfracture, we assessed the gene expression of tight junction‐related proteins within the small intestine and colon. *Ocln* expression significantly increased at day 14 postfracture and remained higher at day 21 postfracture in the PBS control mice; however, *Ocln* gene expression was highly induced at day 3 postfracture in the *B. longum*‐supplemented mice (Figure 3f). Fracture also differentially influenced the gene expression ZO‐1 (*Tjp1*) within the small intestine by significantly increasing the expression at day 14 and 21 postfracture in the PBS control mice compared to baseline expression, while significantly decreasing the expression at day 21 in the *B. longum*‐supplemented mice (Figure 3f). A similar effect was observed on the gene expression of *Jam3*, which was significantly induced at day 21 postfracture in the PBS‐treated mice compared to baseline expression (Figure 3f). *Jam3* gene expression remained unchanged within the small intestine compared to baseline in mice provided *B. longum* during secondary bone repair (Figure 3f). Claudin 2 (*Cldn2*) gene expression was also strongly upregulated within the small intestine of PBS control mice at day 3 postfracture and remained significantly higher until day 21 compared to baseline (prior to fracture) levels (Figure 3f). Conversely, small intestine *Cldn2* gene expression remained unchanged in the *B. longum*‐supplemented mice throughout the fracture healing (Figure 3f). Within the colon, *Ocln* gene expression displayed a dynamic expression pattern in the PBS control mice. Compared to pre‐fracture baseline expression, *Ocln* gene expression was significantly increased at day 7, decreased at day 14 postfracture, and significantly increased at day 21 postfracture (Figure 3g). No differences in colon *Ocln* gene expression were observed in the *B. longum*‐supplemented mice (Figure 3g). Similarly, colon *Tjp1* gene expression was significantly induced at day 7 postfracture in PBS control mice, with levels returning to baseline by day 14 postfracture (Figure 3g). Expression of *Tjp1* was significantly lower at day 14 postfracture in the *B. longum*‐supplemented mice (Figure 3g). Fracture induced a similar increase in the gene expression of *Jam3* and *Muc2* in both groups at day 7 postfracture with levels returning to baseline by day 14 postfracture (Figure 3g). These data indicate that fracture leads to early disruption in intestinal function that persists throughout fracture healing in aged mice, which is attenuated by *B. longum*.

### 3.4 *Bifidobacterium longum* supplementation blunts the systemic inflammatory response to fracture

**FIGURE 4:**
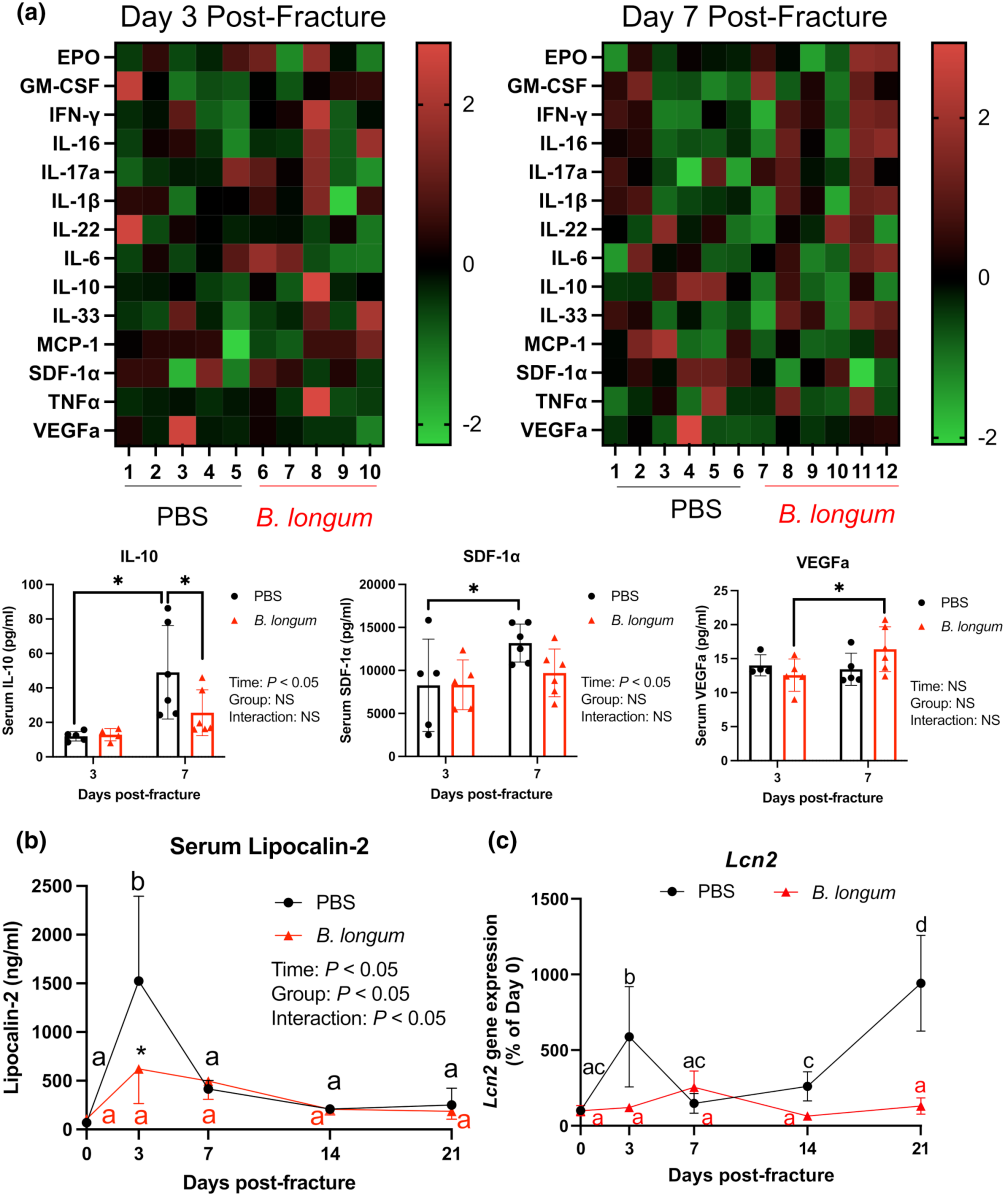
*Bifidobacterium longum* supplementation influenced the fracture‐induced systemic inflammatory response. (a) Heat map of serum cytokines at day 3 (*n* = 4–5/group) and 7 (*n* = 5–6/group) postfracture. Serum IL‐10 and SDF1‐α increased in PBS control mice and VEGFa increased in *B*. longum‐supplemented mice at day 7 compared to day 3 postfracture. *p < 0.05. Each data point represent an independent observation. Outliers were identified using the ROUT method in IL‐10 (excluded value 126 pg/ml) and VEGFa (excluded values 40.3 and 74.7 pg/ml). (b) Fracture induced an increase in serum lipocalin‐2 levels in PBS control mice, which were significantly lower in *B. longum*‐supplemented mice. *p < 0.05 versus PBS (*n* = 4–5/group/timepoint). (c) Fracture induced a significant increase in small intestine lipocalin‐2 (*Lcn2*) gene expression in PBS control mice, but not in *B. longum*‐supplemented mice. Data were assessed using a two‐way ANOVA followed by Sidak's multiple comparison testing. Values of the same color not sharing a common letter are significantly different, *p* < 0.05 (*n* = 4–5/group/timepoint).

Fracture induces a robust local inflammatory response during the initial reactive phase of secondary bone repair that extends to beyond the injured tissue, which is exacerbated in aged animals (Emami et al., 2019; Levy et al., 2006). Serum isolated from mice at day 3 and 7 postfracture, corresponding to the initial inflammatory response, was subjected to a multiplex immunoassay to determine the systemic levels of 16 cytokines and chemokines (Figure 4a and Figure S2). Serum levels of IL‐10 and SDF‐1α were significantly increased from day 3 to day 7 in the PBS control mice, which was not observed in the *B. longum*‐supplemented mice (Figure 4a). *Bifidobacterium longum* also induced an increase in VEGFa levels at day 7 post‐fracture compared to day 3 (Figure 4a). At day 7 post‐fracture, IL‐10 levels were significantly decreased in *B. longum*‐supplemented mice compared to PBS control mice (Figure 4a). Fracture induced a significant acute phase increase (+2141%) in the serum levels of the inflammatory protein lipocalin‐2 at day 3 postfracture in the PBS control mice compared to baseline levels, which was significantly higher than lipocalin‐2 concentrations in the *B. longum*‐supplemented mice (Figure 4b). Within the small intestine, fracture induced a significant increase in lipocalin‐2 (*Lcn2*) gene expression in the control mice at day 3 and 21 postfracture compared to baseline expression (Figure 4c). *Lcn2* gene expression in the small intestine remained unchanged throughout fracture healing in the *B. longum*‐supplemented mice (Figure 4c). These data, when taken together, suggest that *B. longum* dampens the fracture‐induced systemic inflammatory response.

### 3.5 *Bifidobacterium longum* supplementation stabilizes the gut microbiota community structure postfracture

**FIGURE 5:**
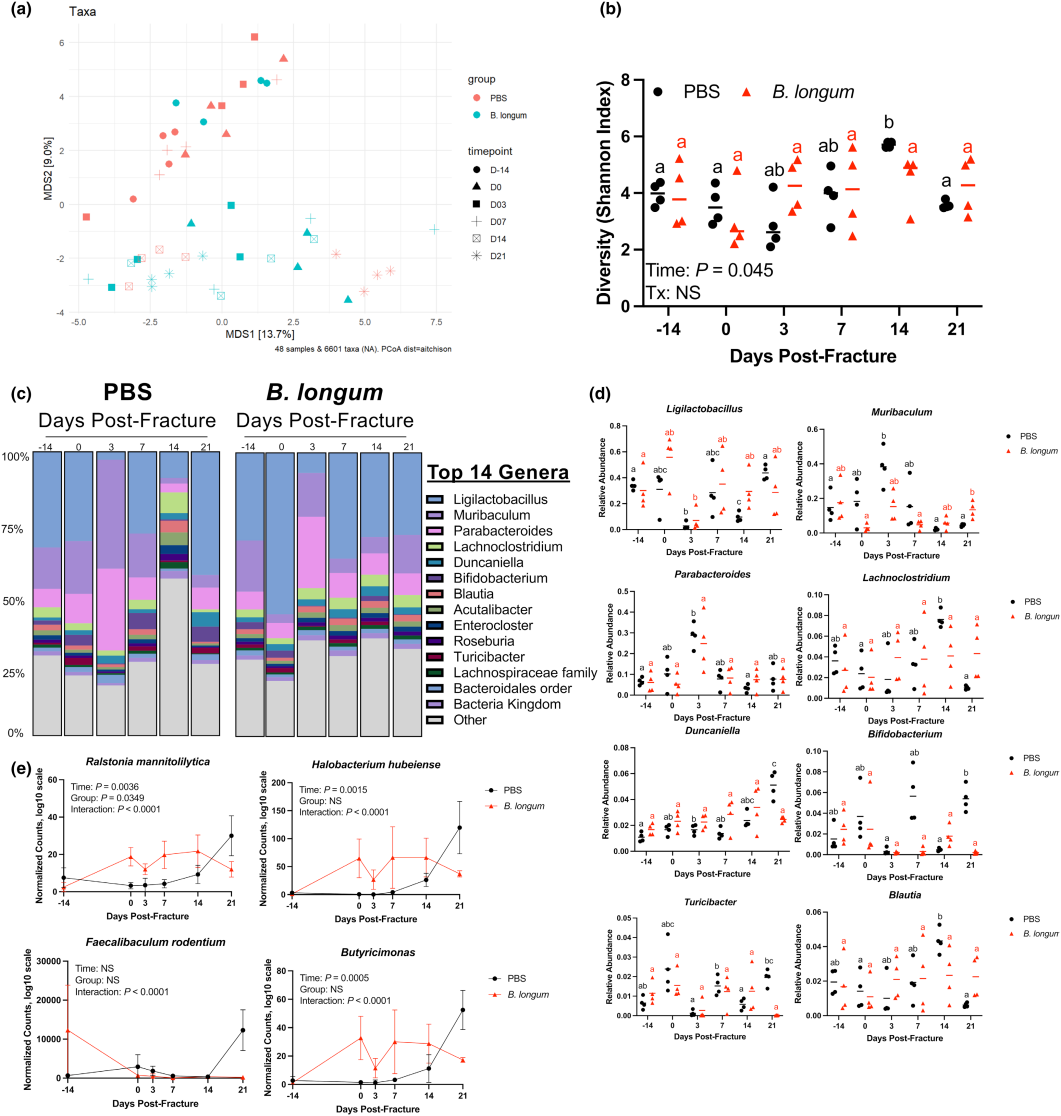
*Bifidobacterium longum* supplementation dampens fracture‐induced dysbiosis. (a) Principal coordinates ordination analyses (PCoA) of fecal beta diversity. (b) Fecal alpha diversity was not affected by fracture in *B. longum*‐supplemented mice. (c) Detailed relative abundance of bacterial taxa at the genera level within fecal samples prior to gavage (day −14), prior to fracture (day 0), and at days 3, 7, 14, and 21 postfracture. (d) Changes in the relative abundances of specific genera throughout fracture healing. (e) *Bifidobacterium longum* supplementation increased and maintained the abundance of *Ralstonia mannitolilytica*, *Halobacterium hubeiense*, *Faecalibaculum rodentium*, and *Butyricimonas* throughout fracture healing. Values of the same color not sharing a common letter are significantly different, *p* < 0.05. Data were assessed using a two‐way ANOVA followed by Sidak's multiple comparison testing. Each data point represent an independent observation (*n* = 4/group/timepoint).

The composition of the gut microbiota is highly malleable and is influenced by traumatic injuries, including fractures (Howard et al., 2017; Li et al., 2022, p.^2022pp; Roberts et al., 2020). Probiotics partly influence host physiology through modulation of the microbiota composition (Tyagi et al., 2018); therefore, we sought to determine whether the *B. longum* would influence the microbiota community structure during convalescence from fracture by first normalizing the microbiomes by repeated exchanges of bedding during the acclimation period to minimize cage effect. Metagenomic sequencing of fecal samples revealed a similar beta diversity (Figure 5a), alpha diversity (Figure 5b), and microbiota community structure in the PBS and *B. longum* cohorts of mice prior to gavage initiation (day ‐14) (Figure 5c), suggesting that the microbiome had been normalized between cages. After 2 weeks of supplementation (day 0 prior to fracture), there was clear separation in the microbial community composition between the PBS control mice and the *B. longum*‐supplemented mice (Figure 5a) and a different taxonomic composition at the genera level between groups (Figure 5c). Analysis of overall microbial community composition changes throughout fracture healing by PERMANOVA on pairwise Aitchison distances revealed a significant effect of treatment (*p* = 0.01, *R* ^2^ = 0.03), timepoint (*p* = 0.001, *R* ^2^ = 0.15), and group:timepoint interaction (*p* = 0.001, *R* ^2^ = 0.15) (Figure 5a). Upon visual inspection of the PCoA plot, the PBS‐supplemented mice clustered together during the early postfracture period; however, at day 14 and 21 postfracture there were shifts in beta diversity to cluster with the *B. longum*‐supplemented mice. Microbial alpha diversity was also significantly increased in the PBS control mice at day 14 postfracture but remained unchanged throughout fracture healing in the *B. longum*‐supplemented mice (Figure 5b). The taxonomic composition at the genera level displayed marked changes throughout the course of fracture healing in the PBS‐supplemented mice that was most evident at day 3 and day 14 postfracture (Figure 5c). This was characterized by a decrease in the relative abundance of *Ligilactobacillus* and increase in the relative abundance of *Muribaculum* and *Parabacteroides* at day 3 postfracture compared to day 0 baseline in the PBS‐supplemented mice (Figure 5d). There was also a significant increase in the relative abundance of *Lachnoclostridium* and *Blautia* at day 14 postfracture and increase in *Duncaniella* at day 21 postfracture in the PBS control mice (Figure 5d). Like the PBS control mice, *B. longum*‐supplemented mice also displayed large changes in the taxonomic composition at day 3 postfracture (Figure 5c). This was largely due to a decrease in the relative abundance of *Ligilactobacillus* (Figure 5d). However, the taxonomic composition returned to baseline by day 7 postfracture and remained unchanged throughout the remaining timepoints for most genera (Figure 5c,d), with the exception of *Muribaculum* that significantly increased at day 21 postfracture in the *B. longum*‐supplemented mice compared to prior to fracture (day 0) (Figure 5d). At the species level, 2 weeks of *B. longum* supplementation (day 0 prior to fracture) increased the abundance of several bacteria including *Ralstonia mannitolilytica*, *Halobacterium hubeiense*, and *Butyricimonas*, which remained unchanged throughout the later timepoints (Figure 5e). In the PBS control mice, the abundance of *Ralstonia mannitolilytica*, *Halobacterium hubeiense*, and *Butyricimonas* did not increase until day 14 postfracture and an increase in *Faecalibaculum rodentium* at day 21 postfracture (Figure 5e). These data demonstrate that fracture rapidly triggers dysbiosis in aged mice, which can be stabilized by *B. longum* supplementation.

## 4 DISCUSSION

Aging is characterized by the gradual deterioration in nearly every organ system. The musculoskeletal system is especially prone to changes caused by aging, and age has long been considered a patient‐specific factor that contributes to adverse healing outcomes (Drissi et al., 2014). Age is also associated with drastic shifts in the composition of the gut microbiota (Castro‐Mejía et al., 2020; Wilmanski et al., 2021), which has been reported to influence bone growth, homeostasis, and repair (Cooney et al., 2020; Roberts et al., 2020). However, the relationship between these age‐related changes in the gut microbiota and fracture healing outcomes is unknown. Here, we demonstrate that modulation the aged gut microbiota using the dietary probiotic supplement *B. longum* can improve healing outcomes in aged mice and attenuate systemic pathologies induced by fractures.

With age there is a natural decline in the regenerative potential of the skeleton, which has been attributed to decreased availability and differentiation capacity of stem cells, heightened inflammation, immunosenescence, and impaired angiogenesis (Clark et al., 2017). Prior studies using 18‐month‐old mice demonstrate that this is a valid healing‐challenged model as indicated by delayed radiographic bridging and reduced callus density at day 14 postfracture compared to young mice (McKenzie et al., 2019; Slade Shantz et al., 2014), as well as forming smaller calluses with less bone (Clark et al., 2020). In our study, *B. longum*‐supplemented mice displayed smaller calluses that contained more bone at day 14 postfracture. These findings indicate that *B. longum* supplementation can accelerate early mineral formation within the callus. Our histologic analyses did not reveal any differences in cartilaginous tissue within the callus, suggesting that this early increase in mineralized tissue is unlikely due to accelerated soft callus formation. Rather, *B. longum* supplementation may promote faster osteoid formation through intramembranous ossification at the callus periphery. This accelerated callus bone formation along with increased cortical bridging in the *B. longum*‐supplemented mice likely promoted the increase in mechanical competence of the fractured bone. Our radiographic scoring and torsion testing results suggest that *B. longum*‐supplementation accelerates the reestablishment of biomechanical properties through bridging of the cortices at fractured site. Fractures are considered healed when bone stability has been restored by the formation of new bone that bridges the cortices (Knox et al., 2021). Mechanistically, *B. longum* has been reported to promote bone health, primarily in the context of ovariectomy‐induced bone loss (Parvaneh et al., 2015; Sapra et al., 2022). These studies demonstrate that supplementation with *B. longum* or its lysate can prevent ovariectomy‐induced bone loss through a mechanism attributed to decreased osteoclastogenesis, increased osteogenesis, and immunomodulation (Montazeri‐Najafabady et al., 2021; Parvaneh et al., 2015; Sapra et al., 2022). Moreover, *B. longum* can induce the gene expression of the osteogenic genes *Bmp‐2* and *Sparc* within the femora of ovariectomized rats (Parvaneh et al., 2015). Fractures in aged mice and rats have reduced expression of BMP‐2 early during fracture healing, which is a potent osteoinductive growth factor (Meyer Jr. et al., 2003; Naik et al., 2009). It is possible that *B. longum* upregulated the expression of BMP‐2 that stimulated bone formation within the callus; however, this remains to be elucidated in future studies. These studies provide some insight into the accelerated callus mineralization response observed in the *B. longum*‐supplemented mice.

We and others have reported that age and simple bone fractures weaken the integrity of the intestinal barrier leading to increased permeability that can permit the translocation of bacterial‐derived virulent factors and drive inflammation (Ahmadi et al., 2020; Roberts et al., 2020). Using probiotics to reinforce the intestinal barrier is promising approach to diminish the systemic pathologies driven by gut leakiness (Li et al., 2016; Ohland & Macnaughton, 2010; Rao & Samak, 2013). We initially reported that the probiotic species *Bifidobacterium adolescentis* could protect against fracture‐mediated dysfunction of intestinal intercellular tight junction genes (Roberts et al., 2020). Similarly, supplementing young mice with the probiotic species *Akkermansia muciniphila* also rescued fractured‐induced gut barrier dysfunction (Liu et al., 2020). Our data indicate that *B. longum* also functions through a comparable mechanism by preventing fracture‐induced intestinal barrier dysfunction in aged mice. This was evidenced by the initial upregulation in gene expression of *Ocln* and stable gene expression of barrier‐forming intestinal tight junction proteins within the small intestine and colon throughout fracture healing in the *B. longum*‐supplemented mice. In the control mice, expression of the tight junction‐related genes only increased during the later stages of bone repair. This suggests that there is compensatory response to fracture‐induced disruptions in intestinal function, which we previously observed in young mice (Roberts et al., 2021). Interestingly, this compensatory response appears to be delayed in the aged mice, with young mice displaying significant increases in tight junction‐associated genes at day 10 postfracture compared to pre‐fracture expression levels (Roberts et al., 2020). There was also a significant early and sustained induction in the expression of *Claudin‐2* (*Clnd2*) within the small intestine of PBS control mice. Claudin‐2 is a pore‐forming claudin that can decrease the transepithelial resistance, thereby increasing permeability (Van Itallie et al., 2008). Inflammatory cytokines (Al‐Sadi et al., 2014; Mankertz et al., 2009; Weber et al., 2010) and certain microbial products, including endotoxin (Liu et al., 2013; Xie et al., 2019) can upregulate the expression of *Cldn2*. The sustained increase in *Cldn2* gene expression may have contributed to the increase in serum endotoxin observed in PBS control mice. Together, these data indicate that fracture leads to barrier dysfunction within the aging gut that can be prevented through *B. longum* consumption.

One intriguing finding is the divergent responses in serum LBP levels between groups. In *B. longum*‐supplemented mice, LBP levels increased significantly after fracture and returned to baseline by day 7 postfracture. However, in the control mice, this initial acute phase response was not observed, with sera levels decreasing at day 14 (−55%) and 21 (−87%) postfracture compared to baseline. LBP is an acute phase protein with a paradoxical role of sensitizing the immune system to endotoxin at low concentrations (Schumann et al., 1990), while high levels of LBP enhance endotoxin neutralization without triggering inflammation (Lamping et al., 1998; Wurfel et al., 1995). It is thought that constitutive levels of LBP facilitate recognition of LPS or gram‐negative infection leading to an immune response, whereas the acute phase increases in LBP function to neutralize LPS thereby attenuating inflammation (Heumann & Roger, 2002). Interestingly, serum endotoxin levels did not differ between groups or between timepoints postfracture until day 21 postfracture, in which there was a significant increase in the PBS control mice. This increase in endotoxin correlated with the lowest sera LBP levels in the PBS control mice. Lower levels of plasma LBP have been associated with increased exposure to Gram‐negative bacteria, the source of endotoxin (Gutsmann et al., 2001). LPS/endotoxin also strongly induces the expression of *Aoah* that encodes for the major enzyme responsible for the inactivation of endotoxin by hydrolyzing acyloxyacyl bonds in the lipid A region of LPS (Munford & Hunter, 1992; Zou et al., 2017). The gene expression of *Aoah* was also strongly upregulated in response to fracture in the control group within the small intestine and colon but did not change in mice receiving *B. longum*. Collectively, these data suggest that fracture leads to dysregulation of endotoxin metabolism, which is not observed with consumption of *B. longum*.

Traumatic injuries can influence the composition and diversity of the gut microbiota (Howard et al., 2017; Li et al., 2022, p.^2022pp; Roberts et al., 2020). We recently reported that consumption of the probiotic species *Bifidobacterium adolescentis* can stabilize the gut microbiota during fracture healing in young mice (Roberts et al., 2020). In this study, we observed a similar effect in aged mice in response to *B. longum* supplementation where both beta diversity and alpha diversity did not change during fracture healing. However, in the control mice, there were significant changes in the microbiota community structure, including an increase in alpha diversity at day 14 postfracture. These data suggest that femoral fracture rapidly leads to dysbiosis that persists throughout healing, which is stabilized with *B. longum*. The increases in lipocalin‐2 expression also come in support of this notion. Lipocalin‐2 is an acute phase protein that chelates siderophores to sequester iron that in turn limits bacterial growth (Asimakopoulou et al., 2016). *Lcn2* expression is upregulated by LPS and has been identified as a sensitive biomarker of intestinal inflammation and dysbiosis (Chassaing et al., 2012; Hsieh et al., 2016; Klüber et al., 2021; Marques et al., 2008). The increases in serum lipocalin‐2 levels observed in our study may reflect both the initial acute phase response to bone injury as well as the changes in microbiome community structure. On the other hand, the changes in gene expression within the small intestine of PBS control mice may initially reflect post‐traumatic intestinal dysfunction and inflammation, whereas the later induction reflects the development of dysbiosis. Importantly, *Lcn2* gene expression did not change in the mice that received *B. longum* throughout fracture healing. Our observation is supported by prior studies demonstrating that *B. longum* supplementation not only reduces fecal lipocalin‐2 but also changes the composition of the microbiota in the highly inflammatory dextran sodium sulfate model of intestinal injury (Singh et al., 2020). Together these data indicate that *B. longum* supplementation can stabilize the community structure of the intestinal microbiota and attenuate fracture‐induced dysbiosis. However, the mechanism through which *B. longum* supplementation influenced the gut microbiota remains unknown. *Bifidobacterium* species, including *B. longum*, can inhibit the abundance of pathobionts within the gastrointestinal tract at the genus level (Singh et al., 2020; Yun et al., 2017). This effect may stem from the production of postbiotics, such as short chain fatty acids, that can inhibit the growth of the opportunistic microbes. For example, *B. longum* can produce metabolites that decrease the pH within the lumen to prevent the growth of the pathogen *Clostridium difficile* (Yun et al., 2017). *Bifidobacterium longum* also supports the growth of other bacteria through cross‐feeding involving the production of postbiotics such as acetate (Falony et al., 2006; Riviere et al., 2015). Moreover, it may also reflect the evolutionary adaptations of *Bifidobacterium* species (Ruiz et al., 2013), which are among the first colonizers of the infant gut, that allow it to outcompete other bacteria for open niches (Xiao et al., 2021).

We also observed significant differences in several bacterial genera and species between the two groups and during healing. It remains unclear if these changes in the gut microbiota contributed to the beneficial effects of *B. longum* on fracture healing or if it stems from shifts in the metabolic activity of the gut microbes. It has been suggested that the beneficial health effects of probiotics largely stem from interactions with the commensal gut microbiota that can influence the collective metabolic activity (Eloe‐Fadrosh et al., 2015; Scott et al., 2015). Many bacteria‐derived bioactive metabolites are associated with improved gut and bone health, including short chain fatty acids, bile acids, and tryptophan and polyamine metabolites (Bellissimo et al., 2021; Lucas et al., 2018; Tyagi et al., 2018; Zhao et al., 2020). It is conceivable that these metabolites would contribute to the observed effects, especially considering the expansion of several short‐chain fatty acid‐producing bacteria genera and species (*Ligilactobacillus*, *Butyricimonas*) in the *B. longum*‐supplemented mice. Additional studies are needed to decipher the contributions of the observed microbial changes to fracture healing in aging and whether their activity as determined by metagenomic, metatranscriptomics, and metabolomics is associated with healing.

Importantly, supplementing aged mice with *B. longum* did not lead to any noticeable detrimental effects, rather *B. longum* attenuated many of the fracture‐induced systemic pathologies. Fracture induced a rapid decrease in body weight in both groups, which is supported by several studies reporting similar responses in rats and mice (Magnusdottir et al., 2021; Roberts & Drissi, 2020; Seifter et al., 1975). Notably, *B. longum* supplementation attenuated this early decrease in body weight. This beneficial effect on post‐traumatic metabolism was also observed in the liver, which weighed significantly more in the *B. longum*‐supplemented mice. This increase in liver weight likely reflected an increase in glycogen stores, which may have been depleted in the PBS control mice due to rapid weight loss (Ma et al., 2021). It is possible that the differences in body weight may partially arise due to differences in food consumption, although we did not measure this in this study. This could have also partly contributed to the beneficial effects of *B. longum* on secondary bone repair, which is a metabolically expensive process (Roberts & Drissi, 2020). The initial increase in serum lipocalin‐2 in the PBS control mice may have also contributed to these differential effects on body weight, as it has anorexigenic effects in mice and nonhuman primates (Mosialou et al., 2017; Petropoulou et al., 2020). Another interesting finding was the decreased spleen weight at day 3 postfracture in the *B. longum*‐supplemented mice compared to PBS controls. Splenic enlargement is driven by increased stimulation through adrenergic and inflammatory signals that are commonly present during recovery from fracture, including LPS (Ajmo Jr et al., 2009; Liverani et al., 2014; Seemann et al., 2017).

Incident fractures also accelerate loss of cancellous bone of the vertebral body, with aged mice displaying a greater loss and prolonged recovery (Emami et al., 2019). In our study of aged mice, we observed a similar response to fracture characterized by a decrease in lumbar spine BMD and BMC and vertebral body microarchitectural indices in the control mice. *Bifidobacterium longum* prevented this fracture‐initiated systemic bone loss response within the lumbar spine. The decrease in BS/BV within the vertebral body at day 35 postfracture suggests that *B. longum* decreased bone resorption, which is the principal driver of post‐traumatic bone loss (Emami et al., 2019). We previously reported a similar effect in young mice given *Bifidobacterium adolescentis* (Roberts et al., 2020), suggesting that several species belonging to the *Bifidobacterium* genera can protect the intact skeleton during recovery from fracture.

There are several limitations of our study. Although, we utilized the inbred C57BL/6JN strain to reduce genetic variability and normalized the microbiota between cages to minimize any cage cohort effects, we are ultimately relying on cross‐sectional data to infer longitudinal effects of aging and *B. longum* supplementation on fracture healing and systemic pathologies. The second is that we only utilized female mice for our analyses. We elected to study female mice due to the higher incidence of fractures in elderly women compared to men (Bergh et al., 2020). Future studies are ultimately warranted to determine if male mice would display a similar or divergent response to *B. longum* supplementation. Finally, our microbiome data examining the changes in taxa and species is observational, and we cannot determine causality from the observed microbial changes. While the observed effects are intriguing, future work will need to experimentally determine the significance of these microbial changes to fracture healing in the context of aging.

In conclusion, this study demonstrates that probiotic modulation of the intestinal microbiota using *B. longum* can improve fracture healing outcomes in aged mice and attenuate systemic pathologies following fracture. These beneficial effects likely stemmed from preservation of intestinal function, dampened inflammation, and inhibition of fracture‐induced dysbiosis. This study highlights the connection between traumatic bone injuries and the gut microbiota during aging, which may represent a novel therapeutic target to accelerate bone repair in the elderly using simple dietary approaches like probiotic supplements.

## AUTHOR CONTRIBUTIONS

JLR and HD conceived of the study. JLR, MG, MC, DBH, and GL performed experiments and collected data. JLR analyzed the data and drafted the manuscript. All authors read and approved the final manuscript.

## CONFLICT OF INTEREST

The authors have no competing conflicts to disclose.

## Supporting information


AppendixS1

## Data Availability

The data that support the findings of this study are available from the corresponding author upon reasonable request.
