# Supplementary material for: Bifidobacterium longum supplementation improves age‐related delays in fracture repair
Source: Aging Cell. 2023 Jan 27;22(4):e13786. doi: 10.1111/acel.13786 (PMC10086533; doi:10.1111/acel.13786)
Supplement: Supplementary file 1 — AppendixS1 [file ACEL-22-e13786-s001.pdf]

Supplementary Table 1. List of primers.

Gene	Sequence (5'→3')	Product Size (bp)	GenBank
Ocln	CCTCCAATGGCAAAGTGAAT<br>CTCCCCACCTGTCGTGTAGT	248	NM_001360538.1
Tjp1	CCACCTCTGTCCAGCTCTTC<br>CACCGGAGTGATGGTTTTCT	249	NM_009386.2
Muc2	GCTCGGAACTCCAGAAAGAAG<br>GCCAGGGAATCGGTAGACAT	104	NM_023566.4
Jam3	CACTACAGCTGGTACCGCAATG<br>CTGGGATTGGCTCTGGAATC	59	NM_023277.4
Cldn2	TCTCAGCCCTGTTTTCTTTGG<br>GGCGAGCAGGAAAAGCAA	55	NM_009902.4
B-actin	AGATGTGGATCAGCAAGCAG<br>GCGCAAGTTAGGTTTTGTCA	125	NM_007393.5
Lcn2	ACGGACTACAACCAGTTCGC<br>CATTGGTCGGTGGGGACAGA	188	NM_008491.1

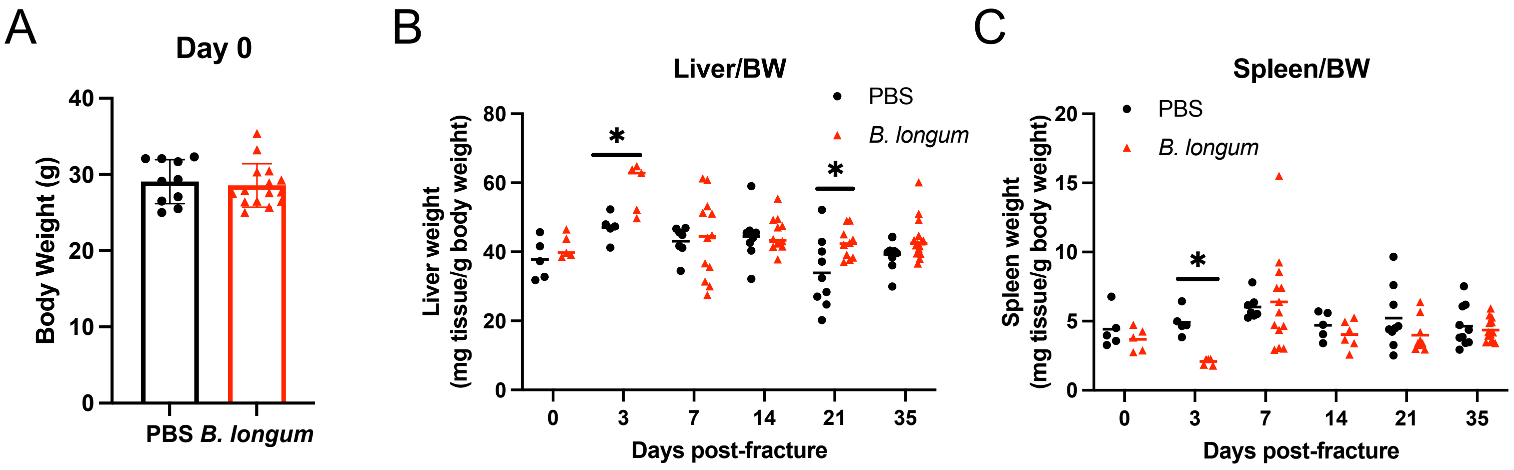

Supplementary Figure 1. No noticeable pathological effects of *B. longum* during recovery from fracture. (A) No difference in body weight between groups after two weeks of pre-supplementation (prior to fracture) with *B. longum*. (B) Liver weight was significantly increased in *B. longum*-supplemented mice at day 3 and day 21 post-fracture. Two-tailed Student's t-test,*$P < 0.05$ vs. PBS. (C) Spleen weight was significantly lower in *B. longum*-supplemented mice at day 3 post-fracture. Two-tailed Student's t-test, *$P < 0.05$ vs. PBS.

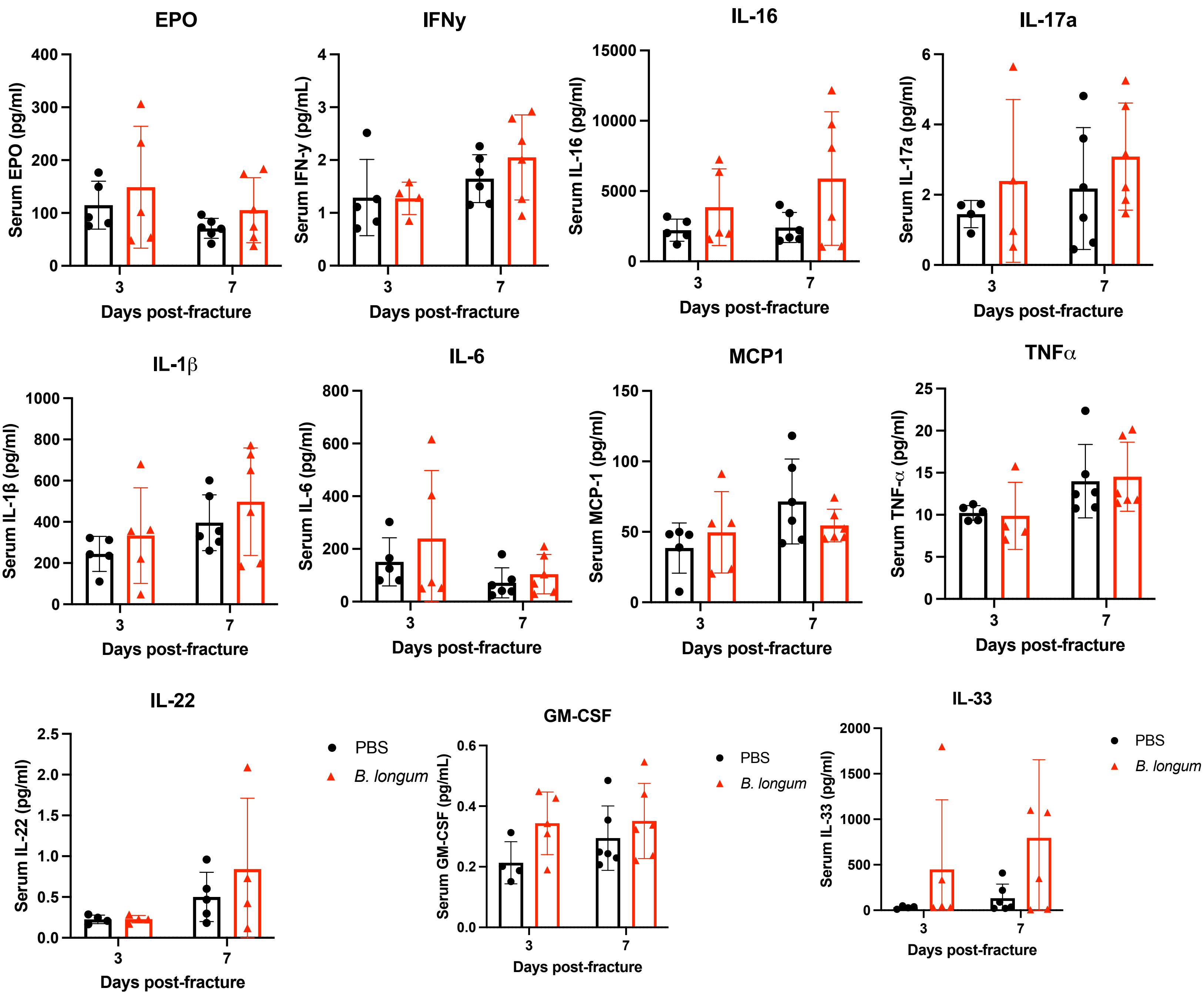

Supplementary Figure 2. Concentration of serum cytokines at day 3 and 7 post-fracture. No significant differences in the concentration of EPO, IFNγ, IL-16, IL-17a, IL-1β, IL-6, MCP1, IL-22, TNFα, GM-CSF, or IL-33 were observed between day 3 and day 7 or between groups.
